# The Food Value of Milk

**Published:** 1906-11-17

**Authors:** 


### The Food Value of Milk.

If one were asked offhand what is the distinguishing characteristic of the best cows' milk, one would promptly reply, "A high percentage of fat." The rapid formation of a full, thick, yellow layer on the surface of milk marks it as rich, nutritious, and wholesome in the eyes of almost everyone, professional and lay alike, and the public backs its opinion by demanding the milk "richest in cream" and scorning milk in which the percentage of fat is low or from which that important constituent has been wholly or partly removed. This popular opinion has influenced cattle-breeding quite unduly. Of all the common varieties of milch-cows, none gives such rich milk as the Jersey; and thus no other breed is so much sought after and valued by the dairy farmer. And yet, in fact, as Dr. J. A. Gilbert shows in an article in the *Medical Record*, this almost universal opinion is not altogether so well founded as to defy criticism. In our earnest search after a fat milk we have probably gone too far. To begin with, the milk which is richest in cream is not, therefore, the most nutritious, for the very simple reason that a rich milk is less easily digested and absorbed than a milk in which the fat percentage is low. As far as its other constituents are concerned, a milk poor in fat is as valuable a food as a milk rich in fat. The fat percentage, the popular standard by which milk is judged, is most variable, while the proportions of the albuminoids, sugars, and salts vary but little in the different samples of milk. In other words, while the energy-producing and heat-giving qualities of the several kinds of milk may be great or little, the valuable proteid ingredients, which go to the building-up of the tissues—the prime property of any food—remain very much the same in all varieties of cows' milk. Thus a "thin" milk is for all purposes, save for energy and heat production, as valuable a food as the so-called "rich" milk. Indeed, it not infrequently happens, as the experimental feeding of young growing animals has shown, that a thin milk may prove, in the long run, more flesh-forming than a rich milk, inasmuch as the former is less liable to induce gastro-enteric disorders. Let us consider what this means. It means, first of all, that the enormous quantity of skim milk produced in this country could be turned to more economical use than the feeding of animals or the manufacture of "ivory" for table knives and piano keys. The despised skim milk is a valuable article of food, capable of supplying many of the wants of the organism, and, from its lightness and digestibility, peculiarly suitable to those whose digestive powers are debilitated. It means, further, that butter-milk, which can be had for the asking almost everywhere in this country, is also a valuable food for men and women, although at present utilised only to feed pigs. Surely, if he is esteemed the greatest benefactor to the race who can grow two grains of corn where only one grew before, in like manner honour should be paid him who rescues a waste product and transforms it into a valued article of a nation's diet.

These considerations lead us, moreover, to doubt how far the popular vote in favour of the Jersey cow is just. It is a well-known fact among cow-feeders that certain Jersey cows give milk so rich in fat that they cannot suckle their own calves. It has been suggested that the difficulty of the digestion of the Jersey milk is due, not only to the relatively high percentage of fat, but also to the fact that the fat globules are individually larger than those in the milk of other breeds. When we remember that the Jersey cow is a delicate animal, highly strung, hard to rear, and difficult to handle, as compared with the more plebeian breeds of Ayrshires or with Holsteins, it becomes plain that, were it not for the altogether artificial and fictitious value set upon the richest milk by the British public, the farmer and dairyman would to a large extent leave the cultivation of the Jersey to the fancy breeder, and rely for commercial purposes upon the more stable and hardy breeds. Of these, probably the Holsteins are the most suitable.

It may seem that this argument runs counter to that experience which has stamped itself upon the laws of this country in such fashion as to make the fat percentage the standard for the estimation of the purity of milk offered for sale. And it may be urged that if a thin milk were legally saleable as "unskimmed milk," watering could never be detected. This objection, of course, leaves out of sight the fact that a watered milk presents a lowered proportion not only of its fat, but also of its other solids. At the same time we are of opinion that public feeling is ripe for a radical change in the laws governing the dairy industry. The public should be protected against unscrupulous dealers, and, in addition to that very simple measure, there should be police supervision to provide against the purveying of a fluid called milk, no doubt, but bearing rather the character of sewage.

